# TETX: a novel nuclear selection marker for *Chlamydomonas reinhardtii* transformation

**DOI:** 10.1186/s13007-015-0064-8

**Published:** 2015-04-15

**Authors:** Sergio A Garcia-Echauri, Guy A Cardineau

**Affiliations:** Centro de Biotecnología-FEMSA, Tecnológico de Monterrey, Monterrey, México; Centro de Agrobiotecnología, Tecnológico de Monterrey, Monterrey, México

**Keywords:** *Chlamydomonas reinhardtii*, Tetracycline resistance, Glass bead transformation, *tet*X

## Abstract

**Background:**

Transformation of microalgae to obtain recombinant proteins, lipids or metabolites of economic value is of growing interest due to low costs associated with culture growth and scaling up. At present there are only three stable nuclear selection markers for the transformation of *Chlamydomonas reinhardtii*, which is the most commonly transformed microalgae, specifically: the aminoglycoside phosphotransferaseses *aph7*and *aphVIII* and the phleomycin resistance *ble* gene. As several microalgae are resistant to some of the antibiotics associated with the mentioned resistance genes, we have developed another alternative, *tet*X, a NADP-requiring Oxidoreductase that hydroxylates tetracycline substrates. We provide evidence that *tetX* can be used to obtain nuclear transformants of *Chlamydomonas reinhardtii*.

**Results:**

We obtained nuclear transformants harbouring the *tet*X gene under the control of beta 2 tubulin or HSP70ARBCS2 promoters at an efficiency of transformation of 3.28 and 6.18 colony forming units/μg DNA respectively. This is the first report of a eukaryotic cell transformed using tetracycline as a selectable marker.

**Conclusions:**

We developed a protocol for the nuclear transformation of *Chlamydomonas reinhardtii* using *tet*X as a selectable marker that confers stable resistance to tetracycline up to 100 μg/mL. We believe *tet*X can be used to transform *Chlamydomonas reinhardtii* chloroplasts, related microalgae and other aerobic organisms sensitive to any tetracycline antibiotic.

## Background

Genetic transformation of microalgae is of growing interest due to its easy growth and low cost scaling up capabilities [1]. *Chlamydomonas reinhardtii* is the most commonly used algae for genetic transformations, however it has been less frequently used to produce nucleus derived recombinant proteins due to transgene silencing [2]. At present, through the use of strong promoter/enhancer sequences like HSP70A and RBCS2 [3,4], introns of RBCS2 [5], and fusions with selectable markers [3,6-8], transgene directed protein production in the cytoplasm has increased to 0.25% of total soluble protein. Although the production levels are still on the order of 10 times lower than comparable production achieved in the chloroplast [1,3], nuclear transformation can produce recombinant proteins that are post-translationally modified and secreted to the exterior of the cell [4].

There are two main mechanisms used to select *C. reinhardtii* nuclear transformants: either generating auxotrophic mutants and then transforming them with the wild-type gene [9] or incorporating a gene that generates resistance to an antibiotic or herbicide. Generating antibiotic resistance is the most frequently used method. Although many microalgae have been transformed, few nuclear selection markers have been expressed stably in the cytoplasm. Specifically, the available selection marker genes are the *aph7* gene from *Streptomyces hygroscopicus* [10]*,* the *Streptomyces rimosus aphVIII* [11] and the *ble* gene from *Streptoalloteichus hindustanus.* Both *aph7* and *aphVIII* confer resistance to different aminoglycoside antibiotics. The *aph7* gene only confers resistance to hygromycin B [10,12], and while a*phVIII* confers paromomycin resistance 2–10 fold higher than the minimum inhibitory concentration (MIC) to *C. reinhardtii*, which is adequate to select transformants, poor results have been seen with G418, kanamycin and neomycin, which require concentrations on the order of 1.2–1.3 fold higher than MIC for selection. As this is inadequate for selection, paromomycin is the agent of choice used to select transformants [11]. Other wild type microalge like *Chlorella sorokiniana*, *Picochlorum* sp. *Botrycoccus braunii*, *Tetraselmis suecica*, *Dunaliella salina* are resistant to paromomycin [13] and probably to other aminoglycoside antibiotics. The *ble* gene generates resistance to phleomycin derived antibiotics [14] such as zeocin, which is the most readily available of that group, however, all the phleomycin antibiotics are expensive. Green algae like *Pseudokirchneriella subcapitata*, the cyanobacteria like *Microcystis aeruginosa*, and *Anabaena* CPB4337 are considered sensitive to tetracycline compounds [15,16]. As there is no reported transformation protocol or selectable marker that generates tetracycline resistance in microalgae or any eukaryote, we pursued the development of such a system for the transformation of the model microalgae *C. reinhardtii*.

There are three main resistance mechanisms that confer tetracycline resistance in bacteria: efflux pumps [17], ribosomal protectors [18] and covalent modifiers [19]. Most proteins that confer resistance to tetracycline used in bacterial transformation are membrane bound effector pumps that export tetracycline to the exterior of the cell. Those could be a challenge to express properly in microalgae and other eukaryotic cells due to their association with the bacterial membrane. Ribosomal protectors are large; for example TetM and TetO are each comprised of 639 amino acid (a.a.) residues (Acc. ADV76307, YP_009080033) and because tetracycline prevents the binding of aminoacyl- tRNA with the bacterial ribosome [20], ribosomal protectors would need to be targeted to the chloroplast and/or mitochondria which contain the homologue to the bacterial ribosome in eukaryotes.

Alternatively, Tetx [21,22] is a 43.7 kDa (388 a.a.) NADP-requiring Oxidoreductase that hydroxylates a broad spectrum of tetracycline substrates resulting in unstable compounds that undergo non-enzymatic decomposition [23]. TetX degrades the following compounds: chlortetracycline, demeclocycline, doxycycline, minocycline, oxytetracycline, tetracycline and tigecycline [23,24]. This is particularly useful in the generation of transformants harboring the *tet*X gene as different organisms have distinct sensitivity to each compound, for example, the protozoan *Toxoplasma gondi* is sensitive to doxycycline and resistant to tetracycline [25], the pathogenic yeast *Candida albicans* is also resistant to tetracycline and sensitive to minocycline and tigecycline [26-28].

Tetracycline resistance used to select genetically modified organisms has been present since the dawn of recombinant DNA technology [29,30], however until this report its use as a selection marker was restricted to bacteria. We designed a synthetic *tet*X gene for expression in the eukaryotic model microalgae *C. reinhardtii* and describe a nuclear transformation protocol for *Chlamydomonas reinhardtii* using *tet*X that generates tetracycline resistance. This is the first report of a genetically modified eukaryote selected with tetracycline. We compare the transformation efficiency of *tet*X under two promoters, beta 2 tubulin and the HSP70ARBCS2, with that of two commonly used selection markers, the *aphVIII* gene controlled by the beta 2 tubulin promoter and the *ble* gene controlled by the RBCS2 promoter. We also performed tetracycline resistance stability assays of transformants grown in the absence of antibiotic.

## Results

### TetX genes

**Figure 1:**
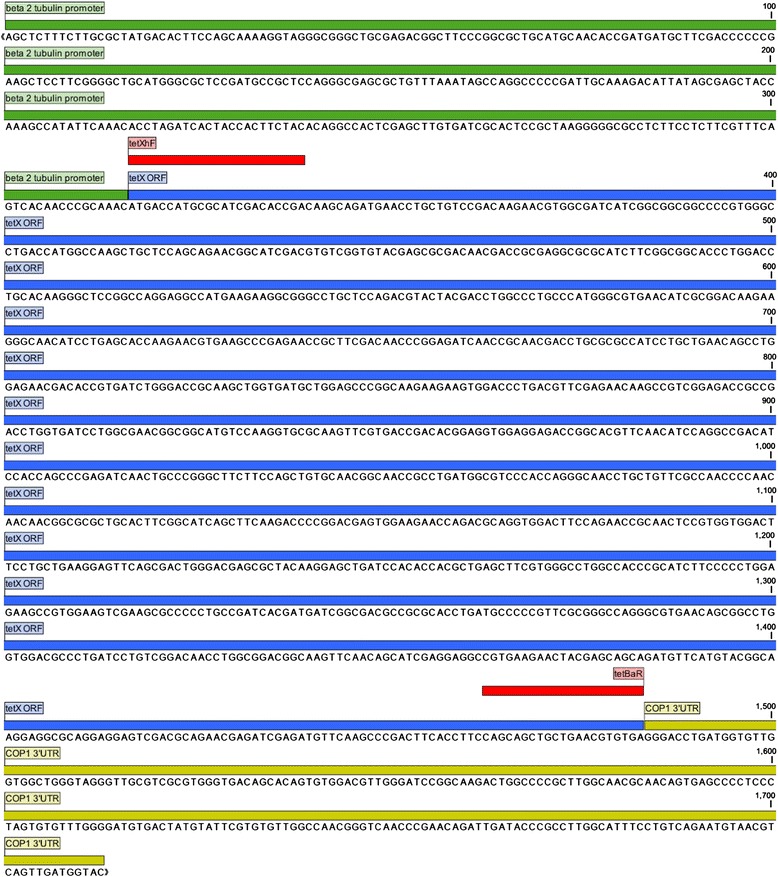
BtetX sequence. The BtetX gene sequence includes the beta 2 tubulin promoter, tetX Open Reading Frame (ORF) and COP 1 3′UTR. In red are primer binding sites for tetXhF and tetBaR.

We synthetized a *tet*X open reading frame codon optimized for expression in *C. reinhardtii* cytoplasm, driven by the beta 2 tubulin promoter [31] and with chlamyopsin1 3′UTR [11] We named this construct BtetX (Figure 1). A second version with *tet*X under HSP70A/RBCS2 enhancer/promoter containing one intron copy of RBCS2 intron 1 and RBCS2 3′UTR was assembled and correct fragments verified by restriction digestion (Data not shown). That plasmid was named AtetX. Plasmid characteristics can be reviewed in Table 1.

**Table 1:** **Plasmids used to transform** ***C. reinhardtii***

**Plasmid**	**Promoter/terminator/#RBCS2 introns**	**Resistance**	**Base pairs/MW (g/mol)**	**TE**	**Source**
AtetX	HSP70A:RBCS2/RBCS2/1	Tetracycline	5238/3236640.1	6.18	This work
BtetX	β-2 tubulin/COP-1/NO	Tetracycline	4445/2746642.9	3.28	This work
pKs-aphVIII	β-2 tubulin/COP-1/NO	Paromomycin	4308/2661961.7	4.51	[38]
psP124S-ble	RBCS2/RBCS2/2	Zeocin	4133/2553770	22.56	[5]

Listing of plasmids used in this work with their corresponding promoter/terminator/number of RBCS2 introns, associated resistance, plasmid size, molecular weight (MW) and Transformation Efficiency (TE).

### TetX transformants were obtained for both constructs

**Figure 2:**
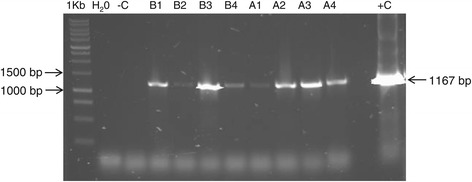
TetX gene presence in AtetX and BtetX transformants. 1% TAE-agarose gel of colony PCR of tetracycline resistant colonies transformed with AtetX (A1-4) or BtetX (B1-4). 1 kb: 1 kb DNA ruler, H_2_O: water was used instead of DNA template, −C: DNA from an untransformed cc-849 strain was used as negative control, +C: AtetX plasmid was used as template.

Transformations with both constructs were carried out and, 8–12 days after plating transformed cells, tetracycline resistant *C. reinhardtii* colonies appeared. Both promoters used to drive *tetX* expression yielded transformant colonies, which indicates the versatility of this system to work with low and high level expression promoters (Figure 2).

### Transformation efficiency

As expected, the HSP70A/RBCS2 enhancer/promoter yielded more colonies than the β-tubulin promoter. In 3 independent transformations the average number of colonies per plate was 20 ± 2.08 and 9 ± 0.57 respectively. The calculated efficiency was 6.18 cfu/μg of DNA for AtetX and 3.28 for BtetX. The efficiency was similar to that of pKS-aphVIII at 4.51 and lower than the *ble* gene with 22.56 (Table 1). We believe the lower efficiency of AtetX compared to the *ble* gene is caused by the addition of an extra RBCS2 intron 1 within the sequence of the *ble* gene, as this extra intron increased the efficiency 3 times compared to a *ble* gene with only one intron [5].

### Effect of light intensity and cell concentration on false positive transformants

As light causes tetracycline degradation, we analyzed the relationship between the appearance of resistant positive or false positive colonies with respect to cell concentration per plate and light intensity (Table 2). When 5 × 10^6^ cells/plate were grown under medium light conditions (17–25 μmoles m^−2^ s^−1^), tetracycline at 15 μg/mL was sufficient to prevent false positives from growing on selection plates. All transformants selected at these conditions were positive. However at concentrations above 5 × 10^6^ cells/plate, and/or light intensity above 27 μmoles m^−2^ s^−1^, false positives appeared as either a lawn or patches of small colonies. However because positive colonies grow faster than negative transformants, they can be easily distinguished and selected from the small colony false positives. Positive transformants can be grown on plates containing tetracycline concentrations up to 100 μg/mL. We also assayed tetracycline concentrations of 25 and 50 μg/mL to select primary transformants, however, at those concentrations the number of positive colonies decreased approximately 75% compared to those obtained with 15 μg/mL (data not shown).

**Table 2:** **Effect of light intensity and cell concentration on false positive appearance**

**Cells/plate**	**Light intensity (μmoles m** ^**−2**^ **s** ^**−1**^ **)**		
	**17**	**24**	**>26**
2.5 × 10^6^	-	-	+
5.0 × 10^6^	-	-	+
1.0 × 10^7^	-	+	+
3.0 × 10^7^	+	+	+

We measured the appearance of false positives on *tet*X transformed *C. reinhardtii* plates when grown at different cell concentrations and light intensities: (−) indicates no false positives, (+) indicates presence of false positives.

### Transformants grown with no antibiotic retained tetracycline resistance

**Figure 3:**
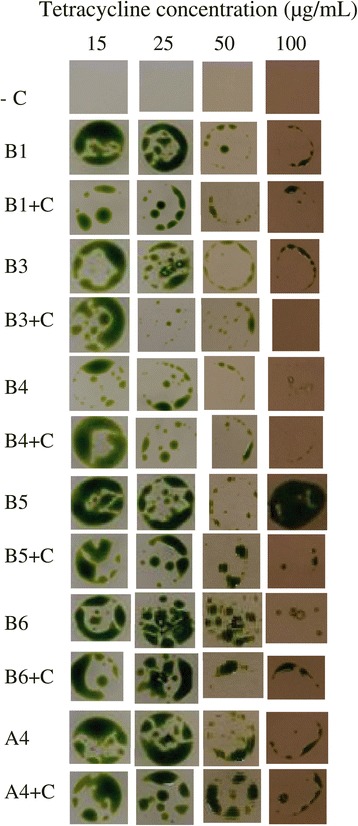
Tetracycline resistance phenotype. BtetX (5 strains) or AtetX (1 strain) positive transformed *C. reinhardtii*, and CC-849 as a negative control, were grown in TAP media with (+C) and without antibiotic selection for 26 cell divisions. A 10 μL droplet containing 10^4^ cells of each strain was plated on TAP plates supplemented with either 15, 25, 50 or 100 μg/mL of tetracycline and incubated with a light intensity of 25 μmoles m^−2^ s^−1^. The tetracycline resistance phenotype is present in all transformed strains. We expected confluent cell growth, however, there appears to be a selection for more resistant cells at higher tetracycline concentrations that grow as patches or along the periphery of the absorbed droplet. Negative controls did not grow at any assayed tetracycline concentration.

Nuclear transgene silencing in *C. reinhardtii* has caused loss of resistance phenotype in half of *aadA* transformants that conferred spectinomycin or streptomycin resistance [32], which is why it is not routinely used for selection. We therefore assayed tetracycline resistance when positive transformed strains expressing the *tetX* gene were grown without antibiotic (Figure 3). Antibiotic resistance was maintained at minimum for 26 divisions, and transformants were also resistant to concentrations up to 100 μg/mL (Figure 3).

### Wild type cell-walled *C. reinhardtii* strain is sensitive to tetracycline

Because we used a cell wall deficient strain for transformation and selection, we tested sensitivity to tetracycline using a wild type (WT) cell-walled strain, CC-124, in order to evaluate more general utility. We therefore grew strain CC-124 in TAP agar plates, under the same light conditions used for transformation, tetracycline concentrations of 15, 25 and 50 μg/mL, and cell concentrations from 0.5 1.0, 2.5 and 5.0 × 10^6^ cells per plate. After a period of 10 days, the time required for transformants to become visible, we found no growth of wild type cells. This suggests that tetracycline uptake at the assayed concentrations is not sufficiently affected by the wild type cell-wall to alter the sensitivity of the cells to tetracycline and that tetracycline selection could work with normal cell-walled wild type cells.

## Conclusions

We have developed a new stable nuclear selection marker for *C. reinhardtii* that confers resistance to tetracycline at up to 100 μg/mL. Although tetracycline is light sensitive and acts in the chloroplast, this was not an obstacle to develop an efficient method to obtain transformants. Compared with hygromycin B, paromomycin and zeocin, tetracycline is by far the least expensive antibiotic (Gold biotechnology, Inc. St. Louis, MO, USA) and provides a reasonable alternative selectable agent for transformation of microalgae. As *tet*X hydrolyzes several tetracycline analogues, [23,33] their use might favor increased light incubation to obtain transformants. We believe that the *tetX* gene produces a versatile tetracycline degrading enzyme, which suggests it could be used to transform the nucleus of other microalgae, as well as the chloroplast or mitochondria of other tetracycline sensitive cells, such as *Saccharomyces cerevisiae* or human HeLa cells that are sensitive to tetracycline concentrations above 10 μg/mL [34,35]. Codon bias of the specific target host and organelle may need to be considered to optimize expression.

## Methods

### Algal strain and growth conditions

Cell wall deficient strain CC-849 of *Chlamydomonas reinhardtii* (Chlamydomonas Resource Center, University of Minnesota) was used in all algal transformation experiments. We chose this strain because it is readily transformed with glass beads or electroporation. Strain CC-124 (Chlamydomonas Resource Center) was used as a control to assay tetracycline sensitivity of WT cell wall strains. All algal strains were grown routinely in TAP media [36] at 25°C with a 16/8 light/dark photoperiod in a growth chamber on top of translucid glass shelves. In front of the shelves, two pairs of Sylvania GRO-LUX 40 W wide spectrum fluorescent light tubes (Osram sylvania ltd. Mississauga, ON, CA) and two OCTRON ECO 32 W fluorescent light tubes (Osram sylvania ltd) placed perpendicular to the shelve provided for light. Plates were placed at 3 to 40 cm from the light source which provided light from 78.6 to 25 μmoles m^−2^ s^−1^. Light intensity was measured with a LI-250A light meter (LI-COR, Lincoln, NE, USA), readings are the sum of 15 second averages from two positions: placing the sensor on top of the glass shelf targeted at the ceiling and at the same place with the sensor targeted towards the floor. To achieve lower than 25 μmoles m^−2^ s^−1^, plates were placed in front of only one pair of Sylvania GRO-LUX 40 W wide spectrum fluorescent light tubes at 30–40 cm from the light source. The lowest light setting: 1.81 μmoles m^−2^ s^−1^, was achieved by placing the plates in the lowest light location in the growth chamber (17 μmoles m^−2^ s^−1^) and covering the plates with two double-layers of gauze.

### Plasmid construction

*E. coli* strains carrying Plasmids pHsp70A/RbcS2-cgLuc [7,37], pSP124S ble cassette [5] and pKS-aphVIII-lox [38] were obtained from the Chlamydomonas Resource Center.

TetX open reading frame [native sequence from *Enterobacteriaceae bacterium;* Genbank: JQ990987] was synthesized *de novo* at GenScript (Piscataway, NJ, USA) with codons optimized for *Chlamydomonas reinhardtii* cytoplasmic expression under the control of constitutive beta 2 tubulin promoter [31] and chlamyopsin1 3′UTR [11]; the plasmid was named Btetx (Figure 1). A second version of the construct was generated by Polymerase Chain Reaction (PCR), amplifying the open reading frame with a Veriti thermal cycler (Applied Biosystems, Foster City, CA, USA) in a 25 μL reaction volume containing 1U of the proof-reading high fidelity (1 error/100,000 bp = 0.001%) enzyme Advantage HD DNA Polymerase (Clontech, Palo alto, USA), 0.1 mM dNTP’s, and 0.25 μM of each oligo tetXhF and tetBaR (Table 3) that carried *Xho*I and *Bam*HI sites in their 5′ends for cloning purposes. The reaction was carried out with an initial denaturation at 98°C 2 min. following 35 cycles of 98°C 10 sec, 70°C 10 sec, 72°C 1 min. with a final 5 min extension at 72°C. The amplicon was digested with *Bam*HI and *Xho*I (New England Biolabs, Ipswich, MA, USA), and cloned into the corresponding sites of plasmid pHsp70A/RbcS2-cgLuc, replacing the luciferase ORF with that of *tet*X. The ligation was transformed into *E. coli* stbl4 (Invitrogen, Carlsbad, CA, USA) generating plasmid AtetX.

**Table 3:** **Oligonucleotides used**

**Name**	**Sequence 5′-3′**
aph8F	CGTGCACTGCGGGGTCGGT
aph8R	CCGCCCCATCCCACCCGC
ble1F	CCGGGTCGCGCAGGGC
ble1R	GCGCCGTTCCGGTGCTCA
tetXhF	**TCTCGAG**ATGACCATGCGCATCGACACCGA
tetBaR	**TGGATCC**TCACACGTTCAGCAGCAGCTGCTG

Listing of oligonucleotides used in this work. Bases in bold indicate recognition sites for restrictions enzymes and accessory bases used for cloning purposes.

### Glass bead transformation

*C. reinhardtii* strain CC-849 was transformed with supercoiled plasmid DNA of Atetx, Btetx, pKS-aphVIII or pSP124S (Table 1) by the glass bead method [39]. Briefly, the algae were grown at 25°C with a 18/6 (light/dark) photoperiod in TAP media to mid-log phase (1–2 × 10^6^ cells/mL), the cells were harvested by centrifugation 5 min. at 5000 × G, the growth medium was removed and fresh TAP was added to achieve a cell concentration of 2 × 10^8^ cells/mL. 300 μL of the cell suspension were placed in a 1.5 mL centrifuge tube containing 0.3 g of sterile 0.4 - 0.6 mm diameter glass beads (Sigma, St. Louis, MO, USA) and 1 × 10^−12^ mols of the desired plasmid DNA. The cell/DNA/glass bead suspensions were vortexed 15 s at maximum power in a VWR mini vortex. The cells were transferred to a glass tube with 5 mL of fresh TAP media and incubated at 25°C overnight with a 8/6 (light/dark) photoperiod. After 14 hours the cells were concentrated by centrifugation at 5,000 × g for 15 minutes and resuspended in TAP media to yield either 3 × 10^7^ cells/mL of pKS-aphVIII and pSP124S transformants and 2.5 × 10^6^, 5 × 10^6^, 1 × 10^7^ or 3 × 10^7^ cells/mL for *tet*X transformants. 300 μL of the cell suspensions were spread on 100 mm diameter and 15 mm depth TAP agar plates supplemented with either 150 μg/mL paromomycin, 20 μg/mL Zeocin or 15 μg/mL tetracycline. Plates were incubated at 25°C with a 18/6 (light/dark) photoperiod in low light (1.81 μmoles m^−2^ s^−1^) for 1 day, and then *tet*X plates were transferred to medium light (25 μmoles m^−2^ s^−1^) while paromomycin and zeocin plates were incubated in high light (78.6 μmoles m^−2^ s^−1^) When colonies appeared, they were streaked on to selective TAP agar plates.

### PCR confirmation of transformants

To verify the gene presence in transformants, colony PCR [40] was performed in a 15 μL reaction volume containing 7.5 μL of GoTaq Green Master Mix (Promega, Madison Wi, USA) , 0.25 μM of each appropriate oligo pair: aph8F, aph8R; ble1F, ble1R; tetXhF, tetBaR (Table 3). An initial 5 minute denaturation at 95°C was performed, followed by 35 cycles of 95°C 20 sec, 60–70°C 15 sec, 72°C 1 min with a final 5 min extension at 72°C. The amplicons were resolved in a 1% TAE-agarose gel stained with Sybr safe (Invitrogen, Carlsbad, CA, USA). Positive colonies confirmed by PCR were counted and the efficiency reported as colony forming units (cfu) per μg of DNA.

### Tetracycline sensitivity resistance assays

Five random strains of BtetX, one of AtetX and one of CC-849 untransformed control were selected from TAP-agar plates and 10^4^ cells of each strain were grown in 2 mL TAP media without tetracycline. Positive controls were grown on TAP agar plates supplemented with 15 μg/mL tetracycline. After five days, cell concentration of the cultures was 1 × 10^7^ cells/mL which corresponds to 26 cell divisions. At that time, cultures of strains grown with or without antibiotic were diluted with TAP media to 1 × 10^6^ cells/mL, and 10 μL (10^4^ cells) of each strain were grown on TAP agar plates containing tetracycline at 15, 25, 50 and 100 μg/μL. After 7 days of growth at a light intensity of 25 μmoles m^−2^ s^−1^ photographs were taken of each plate.

### Wild type *C. reinhardtii* tetracycline sensitivity

*C. reinhardtii* strain CC-124 (wt, mt-) was grown in TAP agar supplemented with tetracycline concentrations of 15, 25 or 50 μg/mL, and cell concentrations from 0.5 1.0, 2.5 and 5.0 × 10 ^6^ cells per plate, incubated for 10 days at a light intensity of 25 μmoles m^−2^ s^−1^.

### TetX plasmid deposit

The TetXA and TetXB transformation plasmids have been deposited with the Chlamydomonas Resource Center, University of Minnesota.

## References

[1] Specht E, Miyake-Stoner S, Mayfield SP (2010). Micro-algae come of age as a platform for recombinant protein production. Biotechnol Lett.

[2] Cerutti H, Johnson AM, Gillham NW, Boynton JE (1997). Epigenetic silencing of a foreign gene in nuclear transformants of Chlamydomonas. Plant Cell.

[3] Rasala BA, Lee PA, Shen Z, Briggs SP, Mendez M, Mayfield SP (2012). Robust Expression and Secretion of Xylanase1 in Chlamydomonas reinhardtii by Fusion to a Selection Gene and Processing with the FMDV 2A Peptide. PLoS One.

[4] Eichler-Stahlberg A, Weisheit W, Ruecker O, Heitzer M (2009). Strategies to facilitate transgene expression in Chlamydomonas reinhardtii. Planta.

[5] Lumbreras V, Stevens D, Purton S (1998). Efficient foreign gene expression in Chlamydomonas reinhardtii mediated by an endogenous intron. Plant J.

[6] Fuhrmann M, Oertel W, Hegemann P (1999). A synthetic gene coding for the green fluorescent protein (GFP) is a versatile reporter in Chlamydomonas reinhardtii. Plant J.

[7] Fuhrmann M, Hausherr A, Ferbitz L, Schödl T, Heitzer M, Hegemann P (2004). Monitoring dynamic expression of nuclear genes in Chlamydomonas reinhardtii by using a synthetic luciferase reporter gene. Plant Mol Biol.

[8] Rasala BA, Barrera DJ, Ng J, Plucinak TM, Rosenberg JN, Weeks DP (2013). Expanding the spectral palette of fluorescent proteins for the green microalga Chlamydomonas reinhardtii. Plant J.

[9] Debuchy R, Purton S, Rochaix JD (1989). The argininosuccinate lyase gene of Chlamydomonas reinhardtii: an important tool for nuclear transformation and for correlating the genetic and molecular maps of the ARG7 locus. EMBO J.

[10] Berthold P, Schmitt R, Mages W (2002). An Engineered Streptomyces hygroscopicus aph 7′′Gene Mediates Dominant Resistance against Hygromycin B in Chlamydomonas reinhardtii. Protist.

[11] Sizova I, Fuhrmann M, Hegemann P (2001). A Streptomyces rimosus aphVIII gene coding for a new type phosphotransferase provides stable antibiotic resistance to Chlamydomonas reinhardtii. Gene.

[12] Blochlinger K, Diggelmann H (1984). Hygromycin B phosphotransferase as a selectable marker for DNA transfer experiments with higher eucaryotic cells. Mol Cell Biol.

[13] Díaz-Santos E, de la Vega M, Vila M, Vigara J, León R (2013). Efficiency of different heterologous promoters in the unicellular microalga Chlamydomonas reinhardtii. Biotechnol Prog.

[14] Stevens DR, Rochaix JD, Purton S (1996). The bacterial phleomycin resistance gene ble as a dominant selectable marker in Chlamydomonas. Mol Gen Genet.

[15] Van der Grinten E, Pikkemaat MG, van den Brandhof E-J, Stroomberg GJ, Kraak MHS (2010). Comparing the sensitivity of algal, cyanobacterial and bacterial bioassays to different groups of antibiotics. Chemosphere.

[16] González-Pleiter M, Gonzalo S, Rodea-Palomares I, Leganés F, Rosal R, Boltes K (2013). Toxicity of five antibiotics and their mixtures towards photosynthetic aquatic organisms: implications for environmental risk assessment. Water Res.

[17] Li X-Z, Nikaido H (2004). Efflux-mediated drug resistance in bacteria. Drugs.

[18] Dönhöfer A, Franckenberg S, Wickles S, Berninghausen O, Beckmann R, Wilson DN (2012). Structural basis for TetM-mediated tetracycline resistance. Proc Natl Acad Sci U S A.

[19] Yu Z, Reichheld SE, Cuthbertson L, Nodwell JR, Davidson AR (2010). Characterization of tetracycline modifying enzymes using a sensitive in vivo reporter system. BMC Biochem.

[20] Chopra I, Roberts M (2001). Tetracycline Antibiotics : Mode of Action, Applications, Molecular Biology, and Epidemiology of Bacterial Resistance Tetracycline Antibiotics: Mode of Action, Applications, Molecular Biology, and Epidemiology of Bacterial Resistance. Microbiol Mol Biol Rev.

[21] Guiney DG, Hasegawa P, Davis CE (1984). Expression in Escherichia coli of cryptic tetracycline resistance genes from bacteroides R plasmids. Plasmid.

[22] Speer BS, Bedzyk L, Salyers AA (1991). Evidence that a novel tetracycline resistance gene found on two Bacteroides transposons encodes an NADP-requiring oxidoreductase. J Bacteriol.

[23] Yang W, Moore IF, Koteva KP, Bareich DC, Hughes DW, Wright GD (2004). TetX is a flavin-dependent monooxygenase conferring resistance to tetracycline antibiotics. J Biol Chem.

[24] Moore IF, Hughes DW, Wright GD (2005). Tigecycline is modified by the flavin-dependent monooxygenase TetX. Biochemistry.

[25] Chang HR, Comte R, Pechère JC (1990). In vitro and in vivo effects of doxycycline on Toxoplasma gondii. Antimicrob Agents Chemother.

[26] Lavarde V, Acar JF, Drouhet E (1975). Effect of minocycline on Candida albicans. “In vitro” study: comparison with tetracycline. Pathol Biol (Paris).

[27] Waterworth PM (1974). The effect of minocycline on Candida albicans. J Clin Pathol.

[28] Ku TSN, Palanisamy SKA, Lee SA (2010). Susceptibility of Candida albicans biofilms to azithromycin, tigecycline and vancomycin and the interaction between tigecycline and antifungals. Int J Antimicrob Agents.

[29] Cohen SN, Chang AC, Hsu L (1972). Nonchromosomal antibiotic resistance in bacteria: genetic transformation of Escherichia coli by R-factor DNA. Proc Natl Acad Sci U S A.

[30] Cohen SN, Chang AC, Boyer HW, Helling RB (1973). Construction of biologically functional bacterial plasmids in vitro. Proc Natl Acad Sci U S A.

[31] Davies JP, Weeks DP, Grossman AR (1992). Expression of the arylsulfatase gene from the β2-tubulin promoter in Chlamydomonas reinhardtii. Nucleic Acids Res.

[32] Cerutti H, Johnson AM, Gillham NW, Boynton JE (1997). A Eubacterial Gene Conferring Spectinomycin Resistance on. Genetics.

[33] Rose WE, Rybak MJ (2006). Tigecycline: first of a new class of antimicrobial agents. Pharmacotherapy.

[34] Garí E, Piedrafita L, Aldea M, Herrero E (1997). A set of vectors with a tetracycline-regulatable promoter system for modulated gene expression in Saccharomyces cerevisiae. Yeast.

[35] Gossen M, Bujard H (1992). Tight control of gene expression in mammalian cells by tetracycline-responsive promoters. Proc Natl Acad Sci U S A.

[36] Harris EH. The Chlamydomonas Sourcebook. A Comprehensive Guide to Biology and Laboratory Use. San Diego, CA: Academic Press. 1989. xiv, 780 pp., illus. 10.1126/science.246.4936.1503-a 17756009

[37] Ruecker O, Zillner K, Groebner-Ferreira R, Heitzer M (2008). Gaussia-luciferase as a sensitive reporter gene for monitoring promoter activity in the nucleus of the green alga Chlamydomonas reinhardtii. Mol Genet Genomics.

[38] Heitzer M, Zschoernig B (2007). Construction of modular tandem expression vectors for the green alga Chlamydomonas reinhardtii using the Cre/lox-system. Biotechniques.

[39] Kindle KL (1990). High-frequency nuclear transformation of Chlamydomonas reinhardtii. Proc Natl Acad Sci U S A.

[40] Cao M, Fu Y, Guo Y, Pan J (2009). Chlamydomonas (Chlorophyceae) colony PCR. Protoplasma.

